# Crystal structure of Pigment Red 254 from X-ray powder diffraction data

**DOI:** 10.1107/S2056989017003309

**Published:** 2017-03-14

**Authors:** Svetlana N. Ivashevskaya

**Affiliations:** a Institute of Geology, Karelian Research Centre, Russian Academy of Sciences, Pushkinskaya 11, 185910 Petrozavodsk, Russian Federation

**Keywords:** powder diffraction, Pigment Red 254, diketo­pyrrolo-pyrrole (DPP) pigments, simulated annealing, Rietveld refinement

## Abstract

The crystal structure of Pigment Red 254 [P.R. 254, C_18_H_10_Cl_2_N_2_O_2_; systematic name: 3,6-bis­(4-chloro­phen­yl)-2,5-di­hydro­pyrrolo­[3,4-*c*]pyrrole-1,4-dione] was solved from laboratory X-ray powder diffraction data using the simulated annealing method followed by Rietveld refinement because the very low solubility of the pigment in all solvents impedes the growth of single crystals suitable for X-ray analysis. The mol­ecule lies across an inversion center. The dihedral angle between the benzene ring and the pyrrole ring in the unique part of the mol­ecule is 11.1 (2)°. In the crystal, mol­ecules are linked *via* N—H⋯O hydrogen bonds, forming chains along [110] incorporating *R*_2_^2^(8) rings.

## Abstract 2

The crystal structure of Pigment Red 254 was successfully solved from laboratory X-ray powder diffraction data by the simulated annealing method followed by Rietveld refinement. The dihedral angle between the benzene and pyrrole rings is 11.1 (2)°. In the crystal, mol­ecules are linked *via* N—H⋯O hydrogen bonds, forming chains along [110] incorporating 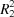
(8) rings.

## Chemical context   

Within the range of diketo­pyrrolo-pyrrole (DPP) pigments presently offered to the market, P.R. 254 plays the most important role (Herbst & Hunger, 2004 ▸), this commercially available type the pigment being widely used in industrial paints, for example for automotive finishes, and plastics which are processed at high temperature. P.R. 254 affords medium shades of red in full shades, while reductions made with a white paint are somewhat bluish red. The pigment demonstrates excellent fastness to organic solvents and weather-fastness, as well as good coloristic and fastness properties. It also shows good hiding power and high tinctorial strength.

The pigment exhibits very low solubility in all solvents, impeding the growth of single crystals suitable for X-ray analyses. Pigments are not dissolved in their application media, but finely dispersed. Consequently the final product properties depend on the crystal structure of the pigments. The crystal structure was successfully solved from laboratory X-ray powder diffraction data using the simulated annealing method followed by Rietveld refinement.
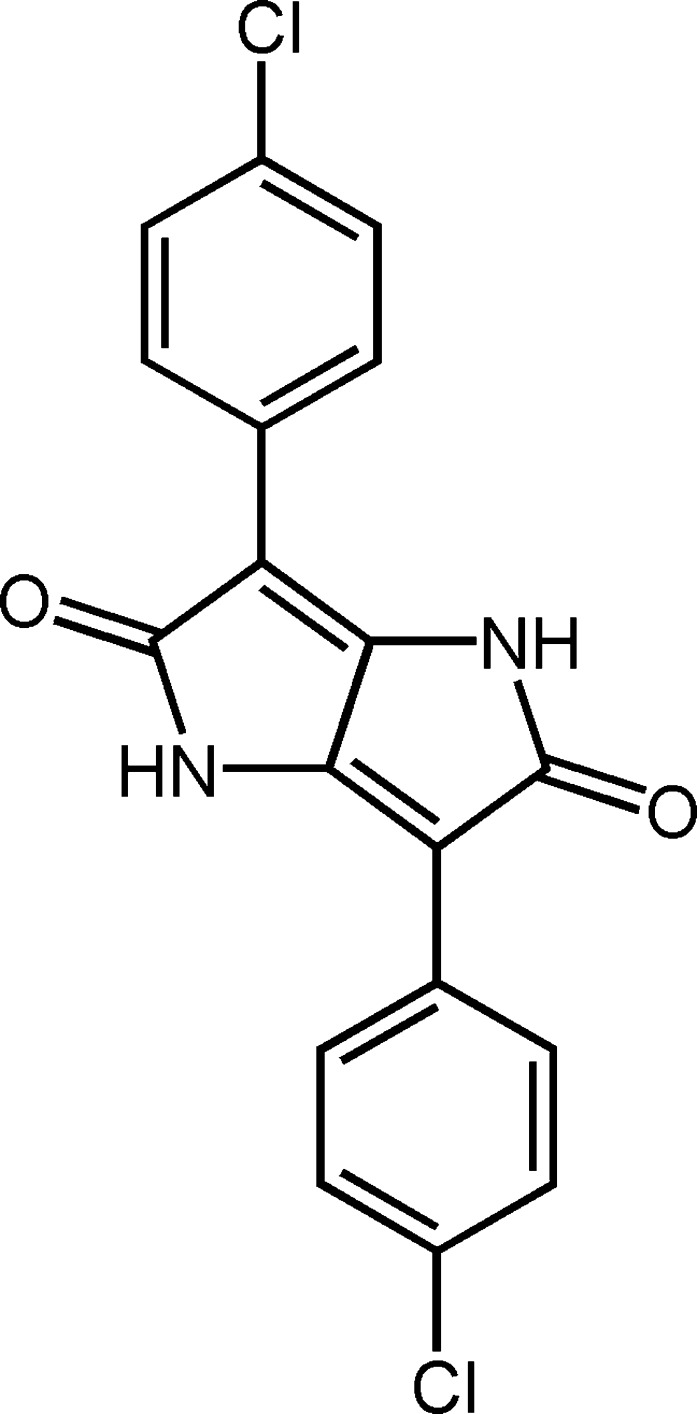


## Structural commentary   

**Figure 1:**
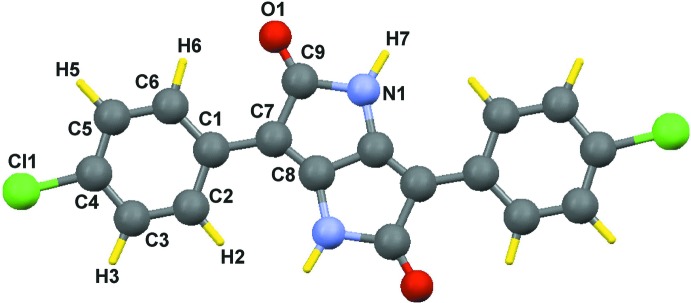
The mol­ecular structure of the title compound. Unlabelled atoms are related by the symmetry code (−*x* + 1, −*y* + 1, −*z* + 1). The atoms are represented by spheres of arbitrary size.

The mol­ecule of the title compound (Fig. 1 ▸) lies across an inversion center. The dihedral angle between the benzene (C1–C6) and pyrrole (N1/C7–C9/C8 rings is 11.1 (2)°. In the crystal, mol­ecules are linked *via* N—H⋯O hydrogen bonds, forming one-dimensional chains along [110] incorporating 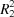
(8) rings.que part of the mol­ecule (C1/C2/C3/C4/C5/C6) and the pyrrole ring [C7/C9/N1/C8/C8(−*x* + 1, −*y* + 1, −*z* + 1)] is 11.1 (2)°. An intra­molecular C—H⋯O hydrogen bond occurs (Table 1 ▸).

## Supra­molecular features   

**Figure 2:**
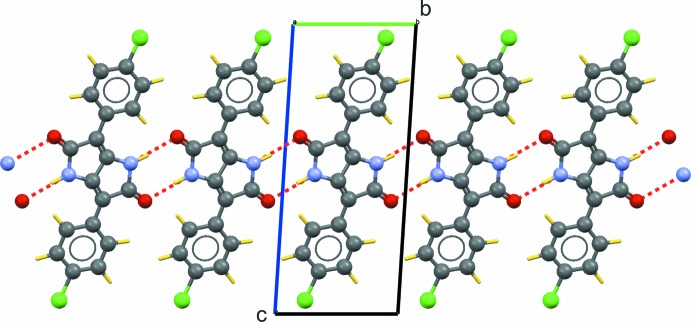
Part of the crystal structure of the title compound (viewed along the *a* axis). Hydrogen bonds are shown as dashed lines.

**Figure 3:**
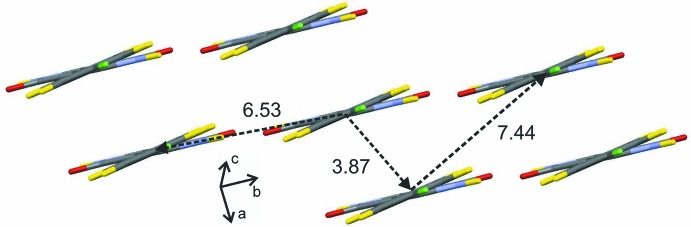
Layered arrangement in the crystal structure of the title compound. Numerical values refer to distances in Å.

In the crystal, mol­ecules are linked *via* N—H⋯O hydrogen bonds, forming chains along [110] incorporating 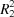
(8) rings (Fig. 2 ▸). In addition, π–π stacking inter­actions between symmetry-related benzene rings with a centroid–centroid distance of 3.871 (2)° connect these chains along [100] (Fig. 3 ▸).

## Synthesis and crystallization   

The technical product P.R. 254 (TR008.052.11-F) supplied by Clariant Produkte (Deutschland) GmbH was taken as is.

## Refinement   

Crystal data, data collection and structure refinement details are summarized in Table 2 ▸. Rietveld refinement was carried out with *TOPAS* (Coelho, 2007 ▸) using all diffraction data. The *TOPAS* input file (including all crystallographic constraints and chemical restraints) was generated automatically by the *DASH*-to-*TOPAS* link.

**Figure 4:**
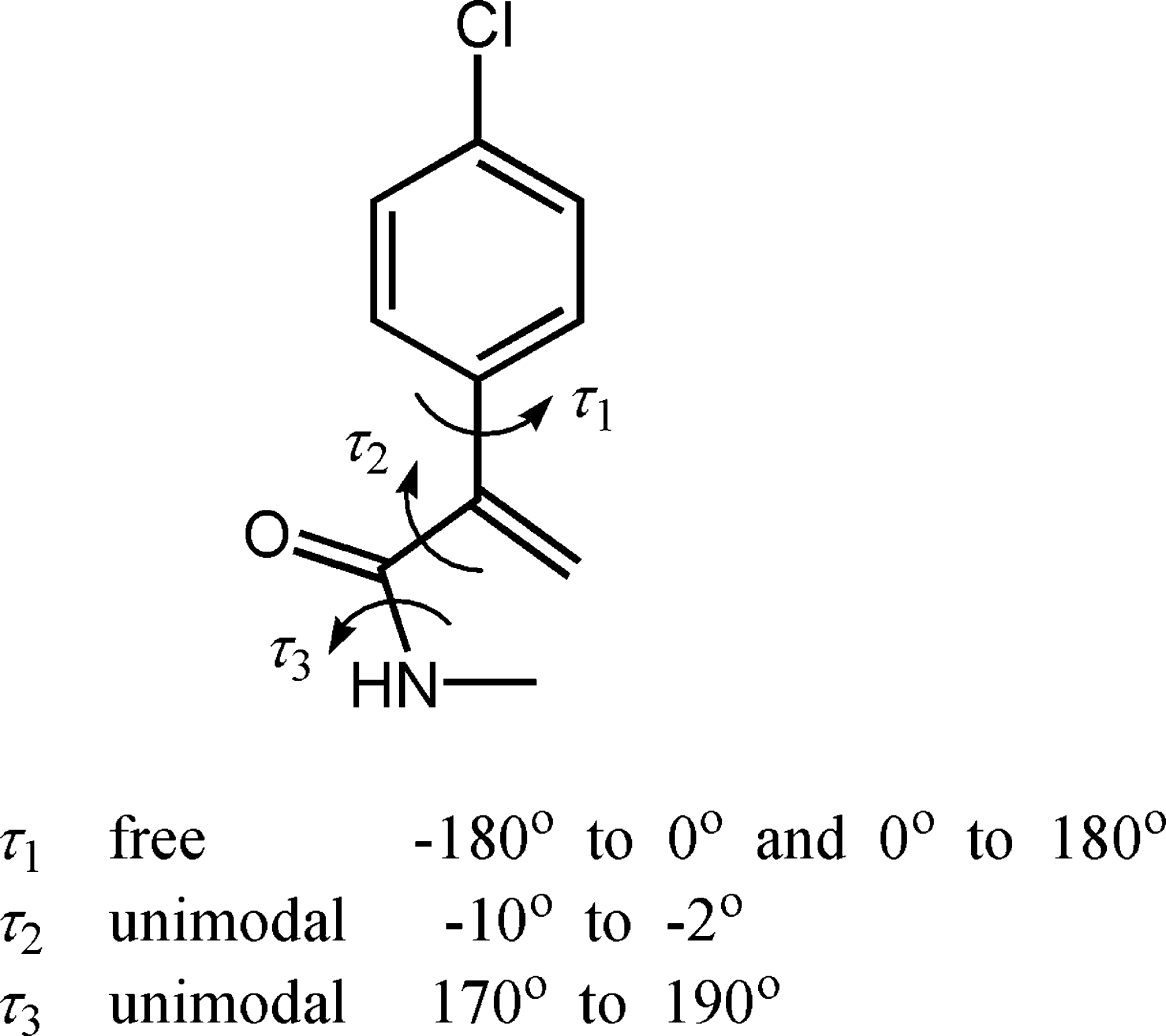
The three flexible torsion angles and their allowed ranges in the structure solution step.

Simulated annealing method (SA) was used to solve the crystal structure from the powder pattern in direct space. The starting mol­ecular geometry was built from known crystal structure of similar compound from the Cambridge Structural Database (CSD; Groom *et al.*, 2016 ▸). The half of the mol­ecule has three flexible torsion angles, which combined with three translational and three orientational degrees of freedom corresponds to a total of nine degrees of freedom. The program *DASH* (David *et al.*, 2006 ▸) was used for structure solution. *DASH* allows the torsion angles to be restricted to inter­vals that significantly reduce the search space. These three flexible torsion angles and their allowed ranges are shown in Fig. 4 ▸. The powder pattern was truncated to a real space resolution of 2.6 Å, which for Cu Kα_1_ radiation corres­ponds to 34.6° in 2θ. The background was subtracted with a Bayesian high-pass filter (David & Sivia, 2001 ▸). The number of SA runs was increased to 50 to get better statistics regarding reproducibility. The background subtraction, peak fitting, Pawley refinement and SA algorithms were used as implemented in the program *DASH*.

Accurate peak positions for indexing were obtained by fitting 20 manually selected peaks with an asymmetry-corrected Voigt function. Indexing was done with the program *DICVOL91* (Boultif & Louër, 1991 ▸). A triclinic unit cell was determined with *M(20)* = 40.7 (de Wolff *et al.*, 1968 ▸), *F(20)* = 82.8 (Smith & Snyder, 1979 ▸). From volume considerations, the unit cell can contain one mol­ecule of P.R. 254 (*Z* = 1). The mol­ecule has an inversion centre, which means the asymmetric unit must consist of a one half of the mol­ecule.

Pawley refinement (Pawley, 1981 ▸) was carried out for refining the background, unit-cell parameters, zero-point error, peak-width and peak-asymmetry parameters. It allows extracting integrated intensities and their correlations. All intensities were refined without reference to a structural model and the result is the best fit that is theoretically possible: *R_wp_* = 13.57, *R_exp _* = 11.20, χ^2^ = 1.467.

**Figure 5:**
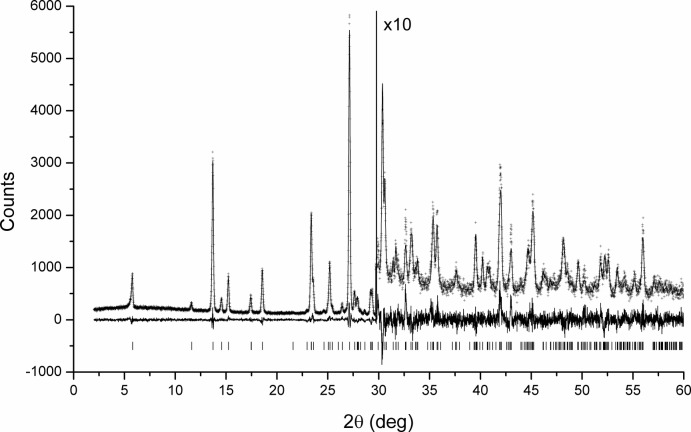
Rietveld plot of P.R. 254. The experimental data points are shown as crosses, the calculated pattern as a solid line and the difference curve as line under the profiles. Tick marks are shown as vertical dashes (laboratory data).

Suitable chemical restraints were applied for all bond lengths, valence angles and the planarity of the aromatic ring systems (including the five-membered condensed system). Anisotropic peak broadening was included to allow the peak profiles to be described accurately. The discrepancies between the observed and the calculated profile appeared to systematically depend on the *hkl* indices of the reflections, indicating preferred orientation in the [001] direction. The March–Dollase formula (Dollase, 1986 ▸) was used. The diffraction profiles and the differences between the measured and calculated profiles are shown in Fig. 5 ▸.

## Supplementary Material

Crystal structure: contains datablock(s) global, I. DOI: 10.1107/S2056989017003309/lh5832sup1.cif

Rietveld powder data: contains datablock(s) I. DOI: 10.1107/S2056989017003309/lh5832Isup2.rtv

Supporting information file. DOI: 10.1107/S2056989017003309/lh5832Isup3.cml

CCDC reference: 1517793

Additional supporting information:  crystallographic information; 3D view; checkCIF report

## Figures and Tables

**Table 1:** Hydrogen-bond geometry (Å, °)

*D*—H⋯*A*	*D*—H	H⋯*A*	*D*⋯*A*	*D*—H⋯*A*
N1—H7⋯O1^i^	0.985 (17)	1.904 (18)	2.884 (5)	173 (2)
C2—H2⋯O1^ii^	1.04 (2)	2.542 (18)	3.489 (6)	151.2 (16)
C2—H2⋯N1^iii^	1.04 (2)	2.55 (2)	3.255 (6)	124.8 (11)
C6—H6⋯O1	1.062 (14)	2.28 (2)	2.959 (6)	119.9 (14)

Symmetry codes: (i) 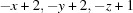; (ii) 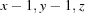; (iii) 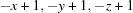.

**Table 2:** Experimental details

Crystal data	
Chemical formula	C_18_H_10_Cl_2_N_2_O_2_
*M*_r_	357.19
Crystal system, space group	Triclinic, *P* 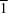
Temperature (K)	293
*a*, *b*, *c* (Å)	3.871 (1), 6.553 (1), 15.292 (1)
α, β, γ (°)	92.773 (3), 94.656 (3), 99.627 (2)
*V* (Å^3^)	380.45 (3)
*Z*	1
Radiation type	Cu *K*α_1_, λ = 1.54056 Å
Specimen shape, size (mm)	Flat sheet, 10 × 10 × 1

Data collection	
Diffractometer	Stoe Stadi-P with linear PSD
Specimen mounting	Sample was prepared between two polyacetate films
Data collection mode	Transmission
Scan method	Step
2θ values (°)	2θ_min_ = 2.00 2θ_max_ = 60.00 2θ_step_ = 0.01

Refinement	
*R* factors and goodness of fit	*R*_p_ = 6.506, *R*_wp_ = 8.578, *R*_exp_ = 7.467, χ^2^ = 1.320
No. of parameters	115
No. of restraints	44
H-atom treatment	All H-atom parameters refined

Computer programs: *WINX^POW^* (Stoe & Cie, 2004 ▸), *TOPAS-Academic* (Coelho, 2007 ▸), *DASH3.1* (David *et al.*, 2006 ▸) and *Mercury* (Macrae *et al.*, 2008 ▸).

## supplementary crystallographic information

### Crystal data

**Table:**

C_18_H_10_Cl_2_N_2_O_2_	*V* = 380.45 (3) Å^3^
*M**_r_* = 357.19	*Z* = 1
Triclinic, *P*1	*D*_x_ = 1.57 Mg m^−^^3^
*a* = 3.871 (1) Å	Cu *K*α_1_ radiation, λ = 1.54056 Å
*b* = 6.553 (1) Å	*T* = 293 K
*c* = 15.292 (1) Å	Particle morphology: powder
α = 92.773 (3)°	red
β = 94.656 (3)°	flat_sheet, 10 × 10 mm
γ = 99.627 (2)°	

### Data collection

**Table:**

Stoe Stadi-P with linear PSD diffractometer	Data collection mode: transmission
Radiation source: sealed X-ray tube	Scan method: step
Primary focussing, Ge 111 monochromator	2θ_min_ = 2.00°, 2θ_max_ = 60.00°, 2θ_step_ = 0.01°
Specimen mounting: sample was prepared between two polyacetate films	

### Refinement

**Table:**

Refinement on *I*_net_	115 parameters
Least-squares matrix: full with fixed elements per cycle	44 restraints
*R*_p_ = 6.506	0 constraints
*R*_wp_ = 8.578	All H-atom parameters refined
*R*_exp_ = 7.467	Weighting scheme based on measured s.u.'s *w* = 1/σ[Y_obs_)^2^
5800 data points	(Δ/σ)_max_ = 0.001
Excluded region(s): none	Background function: Chebyshev function with 20 terms
Profile function: fundamental parameters	Preferred orientation correction: March–Dollase formula (Dollase, 1986), (001) direction

### Fractional atomic coordinates and isotropic or equivalent isotropic displacement parameters (Å^2^)

**Table:**

	*x*	*y*	*z*	*U*_iso_*/*U*_eq_	
C1	0.8418 (7)	0.4738 (6)	0.6911 (3)	0.05373	
C2	0.7516 (10)	0.2631 (7)	0.7088 (3)	0.05373	
C3	0.8543 (9)	0.1967 (6)	0.7905 (3)	0.05373	
C4	1.0452 (8)	0.3351 (7)	0.8528 (3)	0.05373	
C5	1.1403 (8)	0.5402 (7)	0.8377 (3)	0.05373	
C6	1.0380 (9)	0.6065 (7)	0.7573 (3)	0.05373	
C7	0.7380 (7)	0.5404 (6)	0.6038 (3)	0.05373	
C8	0.4818 (10)	0.5527 (8)	0.4587 (4)	0.05373	
C9	0.8702 (6)	0.7478 (4)	0.5715 (2)	0.05373	
H2	0.606 (5)	0.157 (2)	0.6614 (15)	0.06447	
H3	0.796 (4)	0.039 (2)	0.8095 (13)	0.06447	
H5	1.286 (4)	0.644 (2)	0.8892 (16)	0.06447	
H6	1.113 (4)	0.767 (2)	0.7484 (14)	0.06447	
H7	0.774 (5)	0.879 (2)	0.4529 (17)	0.06447	
O1	1.0679 (7)	0.8859 (6)	0.6108 (3)	0.05373	
N1	0.7025 (7)	0.7478 (5)	0.4806 (3)	0.05373	
Cl1	1.1601 (7)	0.2385 (4)	0.9528 (3)	0.05373	

### Geometric parameters (Å, º)

**Table:**

C1—C2	1.411 (6)	C3—H3	1.08 (2)
C1—C6	1.388 (5)	C5—H5	1.07 (2)
C1—C7	1.470 (6)	C6—H6	1.06 (1)
C2—C3	1.392 (6)	C9—O1	1.186 (4)
C3—C4	1.362 (6)	C9—N1	1.485 (5)
C4—C5	1.369 (6)	N1—C8	1.422 (5)
C5—C6	1.374 (6)	N1—H7	0.98 (2)
C7—C9	1.492 (5)	C4—Cl1	1.736 (6)
C2—H2	1.04 (2)		
			
C2—C1—C6	117.3 (4)	C2—C3—H3	125 (1)
C2—C1—C7	119.3 (3)	C4—C3—H3	115 (1)
C6—C1—C7	123.4 (3)	C6—C5—H5	122 (1)
C1—C2—C3	120.0 (3)	C4—C5—H5	119 (1)
C1—C6—C5	122.5 (4)	C7—C9—O1	126.8 (4)
C1—C7—C9	124.4 (3)	C7—C9—N1	106.4 (2)
C2—C3—C4	119.8 (4)	C9—N1—C8	108.8 (4)
C6—C5—C4	118.5 (4)	O1—C9—N1	126.8 (3)
C3—C4—C5	121.8 (4)	C3—C4—Cl1	116.6 (4)
C1—C2—H2	120 (1)	C5—C4—Cl1	121.6 (3)
C3—C2—H2	119.7 (9)	C9—N1—H7	113 (1)
C1—C6—H6	121 (1)	C8—N1—H7	138 (1)
C5—C6—H6	116 (1)		

### Hydrogen-bond geometry (Å, º)

**Table:**

*D*—H···*A*	*D*—H	H···*A*	*D*···*A*	*D*—H···*A*
N1—H7···O1^i^	0.985 (17)	1.904 (18)	2.884 (5)	173 (2)
C2—H2···O1^ii^	1.04 (2)	2.542 (18)	3.489 (6)	151.2 (16)
C2—H2···N1^iii^	1.04 (2)	2.55 (2)	3.255 (6)	124.8 (11)
C6—H6···O1	1.062 (14)	2.28 (2)	2.959 (6)	119.9 (14)

Symmetry codes: (i) −*x*+2, −*y*+2, −*z*+1; (ii) *x*−1, *y*−1, *z*; (iii) −*x*+1, −*y*+1, −*z*+1.
